# Bank-Firm Credit Network in Japan: An Analysis of a Bipartite Network

**DOI:** 10.1371/journal.pone.0123079

**Published:** 2015-05-01

**Authors:** Luca Marotta, Salvatore Miccichè, Yoshi Fujiwara, Hiroshi Iyetomi, Hideaki Aoyama, Mauro Gallegati, Rosario N. Mantegna

**Affiliations:** 1 Dipartimento di Fisica e Chimica, Università di Palermo, Palermo, Italy; 2 Graduate School of Simulation Studies, The University of Hyogo, Kobe, Japan; 3 Department of Mathematics, Niigata University, Niigata, Japan; 4 Graduate School of Sciences, Kyoto University, Kyoto, Japan; 5 Dipartimento di Scienze Economiche e Sociali, Università Politecnica delle Marche, Ancona, Italy; 6 Center for Network Science and Department of Economics, Central European University, Budapest, Hungary; Cinvestav-Merida, MEXICO

## Abstract

We investigate the networked nature of the Japanese credit market. Our investigation is performed with tools of network science. In our investigation we perform community detection with an algorithm which is identifying communities composed of both banks and firms. We show that the communities obtained by directly working on the bipartite network carry information about the networked nature of the Japanese credit market. Our analysis is performed for each calendar year during the time period from 1980 to 2011. To investigate the time evolution of the networked structure of the credit market we introduce a new statistical method to track the time evolution of detected communities. We then characterize the time evolution of communities by detecting for each time evolving set of communities the over-expression of attributes of firms and banks. Specifically, we consider as attributes the economic sector and the geographical location of firms and the type of banks. In our 32-year-long analysis we detect a persistence of the over-expression of attributes of communities of banks and firms together with a slow dynamic of changes from some specific attributes to new ones. Our empirical observations show that the credit market in Japan is a networked market where the type of banks, geographical location of firms and banks, and economic sector of the firm play a role in shaping the credit relationships between banks and firms.

## Introduction

One key economic questions in the description of markets is whether a market is characterized by an underlying networked structure [1]. In fact, in the presence of a networked structure, the price formation process is affected by the underlying network. In this paper we empirically investigate, with tools of network science, the networked structure of the Japanese credit market over a period of time of more than 30 years. The fact that we investigate the credit market for such a long period of time allows us to show that a networked market is present over the years and that its structure slowly evolve over time.

In our approach we consider the Japanese credit market as a bipartite network of banks and firms. Bipartite networks are quite common in complex systems. Classic examples are networks of actors and movies, board members and companies, authors and scientific papers, etc. The customary investigation of bipartite networks is done by performing a one-mode projection for one or both of the two sets of vertices [2]. This approach has been quite successful in the investigation of many bipartite complex systems. However, one-mode projection implies a certain degree of information loss that might prevent, for example, a characterization involving information about direct relationships between nodes of the two sets. In this paper, we investigate the bipartite network of credit relationships established between banks and firms traded at the stock exchanges and over-the-counter markets of Japan. In our investigations we detect and characterize communities of banks and firms that were present in the Japanese credit market during the period of time from 1980 to 2011.

Complex systems can be monitored over long periods of time. The analysis and modeling of these systems in terms of networks can be done by considering the network connections observed for the whole time period and/or by analyzing the network at successive time intervals as, for example, daily, weekly, monthly or yearly time intervals. Here we investigate the bipartite network of credit relationships yearly from 1980 to 2011 by obtaining 32 distinct credit networks. For each year we obtain the credit network and its community structure by using Barber’s BRIM (bipartite recursively induced modules) algorithm [3].

To investigate the time evolution of the communities detected for different years, and, more generally, the time evolution of the underlying networked structure, we need a method that tracks the dynamics of the different communities over time. This method is needed because we are interested into the dynamics of the communities and due to an unavoidable level of uncertainty related to the statistical nature of the community detection process. In this paper, we propose a method which is able to track the time evolution of communities obtained at successive periods of time. The method is more general than just using transition probabilities and it is based on a statistical test which is robust with respect to the heterogeneity of the size of communities and therefore works for communities of different sizes. Our statistical validation procedure of the time evolution of communities allow us to track efficiently the evolution of the communities over time.

The dynamics of the networked structure of the system is investigated by characterizing the communities obtained for different years in terms of the over-expression of attributes of banks and firms concerning (i) the regional location of firms, (ii) economic sectors of firms, and (iii) the types of banks. The statistical validation of the over-expressed attributes is done by using the method presented in [4]. This method uses a multiple hypothesis test correction procedure. With our approach we detect layers of networked credit relationships [1] that have been present in Japan for many years. These layers of credit relationships are characterized by specific types of banks, by firms located in the same or closely related geographical regions and by firms preferentially involved in specific economic sectors. The networked structure of the credit market is slowly evolving over time and the time scale for this evolution is of the order of several years.

The paper is organized as follows. In Subsection “Dataset” of Material and Methods we briefly discuss our dataset. Subsection “Community detection in bipartite networks” discusses community detection in the bipartite network of the Japanese credit market. Subsection of Results entitled “Time evolution of communities” introduces a method used to track the time evolution of communities detected in networks and obtained for successive time periods. Subsection “Over-expression of attributes” presents the empirical results obtained in the characterization of the over-expression of attributes of banks and firms in each community over the years and in Section “Discussion” we draw our conclusions.

## Materials and Methods

### Dataset

Our dataset is based on a survey of firms quoted in the Japanese stock exchange markets (Tokyo, Osaka, Nagoya, in the order of market size) and in Japanese over-the-counter (OTC) markets. The data were compiled from the firms’ financial statements and survey by Nikkei Media Marketing, Inc. in Tokyo, and are commercially available [5]. The survey is done to complement self-reporting, i.e. listed firms may not report in the financial statements in relatively few cases. Yet, according to the Nikkei, the survey often works to obtain the necessary information, although there is no official figures for the remaining missing data. The dataset includes the information about each firm’s borrowing obtained from financial institutions. Specifically, the dataset reports the amounts of borrowing and their classification into short-term and long-term borrowings. Long-term borrowings are considered all contracts exceeding 1 year. We examined the period 1980 to 2011, which is a time period of more than three decades. The analysis is performed yearly, and each yearly network is constructed from the dataset by using the financial statements of the considered calendar year. Since 1996 the dataset includes also OTC markets and/or JASDAQ (the present OTC market). In other studies firms of the over-the-counter market have been excluded to focus on publicly quoted firms. In the present study we investigate all firms which are present in the database. Our choice is motivated by the fact that the network science methods we use in our analysis are only weakly dependent by the size of the system and by the type of heterogeneity of banks, firms and their attributes. Below we verify that indeed our analysis is robust with respect to the enlargement of the dataset by also presenting a separate analysis for the listed and OTC firms for the period 1996–2011.

The number of banks of the database changes year by year by selecting the partition associated with the highest modularity among the 20 performed runs. It was 225 in 1980, remained approximately constant until 2001 and then decreased to 166 in 2011. The number of firms was first increasing from the value of 1414 in 1980 to the value of 3034 in 2006 and then decreasing to the value of 2706 in 2011. The number of firms increased from 1802 in 1995 to 2602 in 1996 in the presence of the first inclusion of the OTC firms in the database. During the same years the number of banks increased from 219 to 226. The density of links in the bipartite network defined as number of observed links over number of potential links was on average decreasing from the value of 0.0867 in 1980 to the value of 0.0398 in 2011. The variation of the density of links was not too large during the first inclusion of the OTC firm. In fact the density of links decreased from 0.0721 to 0.0601 from 1995 to 1996. In the supporting information we provide a summary statistics for the banks and firms of our database. Specifically, for each year we provide the occurrence of the different type of attributes characterizing banks and firms (see S1–S3 Tables). Starting from 1996, the summary statistics is also provide separately for listed and OTC firms (see S4–S9 Tables). From the analysis of the files it can be concluded that the distribution of the geographical and economic attributes of the firms is pretty similar for the listed and the OTC firms. In fact the Pearson correlation coefficient between the vector of the average percent of firms belonging to the different prefectures of listed and OTC firms is 0.969 indicating an almost overlapping distribution of attributes. In the case of the distribution of economic sectors the correlation coefficient is 0.730, which is indicating a rather strong correlation also in this case. In the supporting information we are also providing files where we report on the degree of banks and firms over the years (see S10–S15 Tables). Specifically, the files contain the maximum, minimum, average and standard deviation of the degree. The degree distribution of banks is strongly skewed. In fact, the standard deviation is more than the average value whereas the degree distribution of firms is approximately exponential having the standard deviation approximately the same value of the average value.

The Japanese credit market has been previously analyzed by considering one-mode projected networks [6], an eigenvalue problem determined by the weight of the credit network [7], and, as in the present paper, in terms of communities detected directly on the bipartite network [8].

Concerning financial institutions, commercial banks are long-term, city, regional (primary and secondary), trust banks, insurance banks and government-related financial institutions including credit associations but excluding the Bank of Japan. We remark that failed banks are included until the year of failure, and that merger and acquisition of banks are processed consistently to identify surviving banks. For quoted firms, those who are active in each investigated calendar year are all included even if they failed later during the considered years.

### Community detection in bipartite networks

In our bipartite network a link is present between bank *i* and firm *j* when a credit relation (short and/or long) is present between *i* and *j*. Links are described by a binary variable (just indicating the presence or absence or a credit relationship), i.e., in the present investigation the bipartite network is an unweighted network.

Community detection in large and dense bipartite networks has been considered in the past years by several authors [3, 9–12] and it is still a topic of current research [13, 14]. As for unipartite networks, community detection in bipartite networks is performed by using different approaches and different fitness measures. One widely used fitness measure is the modularity [2], i.e., the measure of the fraction of links in the network connecting vertices of the same community minus the expected value of the same quantity in the corresponding configuration model. The modularity was introduced for unipartite networks in [2] and it was generalized and adapted to bipartite networks in [3, 9–11]. The algorithms based on the generalization to the bipartite case of the modularity [3, 9–11] differ among them with respect to the type of generalization. They also differed with respect to the type of communities obtained. Specifically, in Guimera et al [9] only communities with nodes of the same type are obtained. This is also the case for the algorithms of Murata [10] and Suzuki and Wakita [11] although in their case a one-to-many correspondence of each community of a specific type of nodes can be obtained.

The algorithm of Barber [3] is the only one providing communities that are composed by nodes of both types and are providing a one-to-one correspondence between a group of nodes of one set and a group of nodes of the other set. In the present study, we are explicitly interested in investigating the one-to-one correspondence of groups of banks with related groups of firms. For this reason we have decided to use Barber’s algorithm.

In our analysis we have repeated the application of BRIM community detection algorithm a number of times for each year we investigate. Specifically, for each investigated year in each run we apply the algorithm 100 times and we perform 20 independent runs.

**Fig 1:**
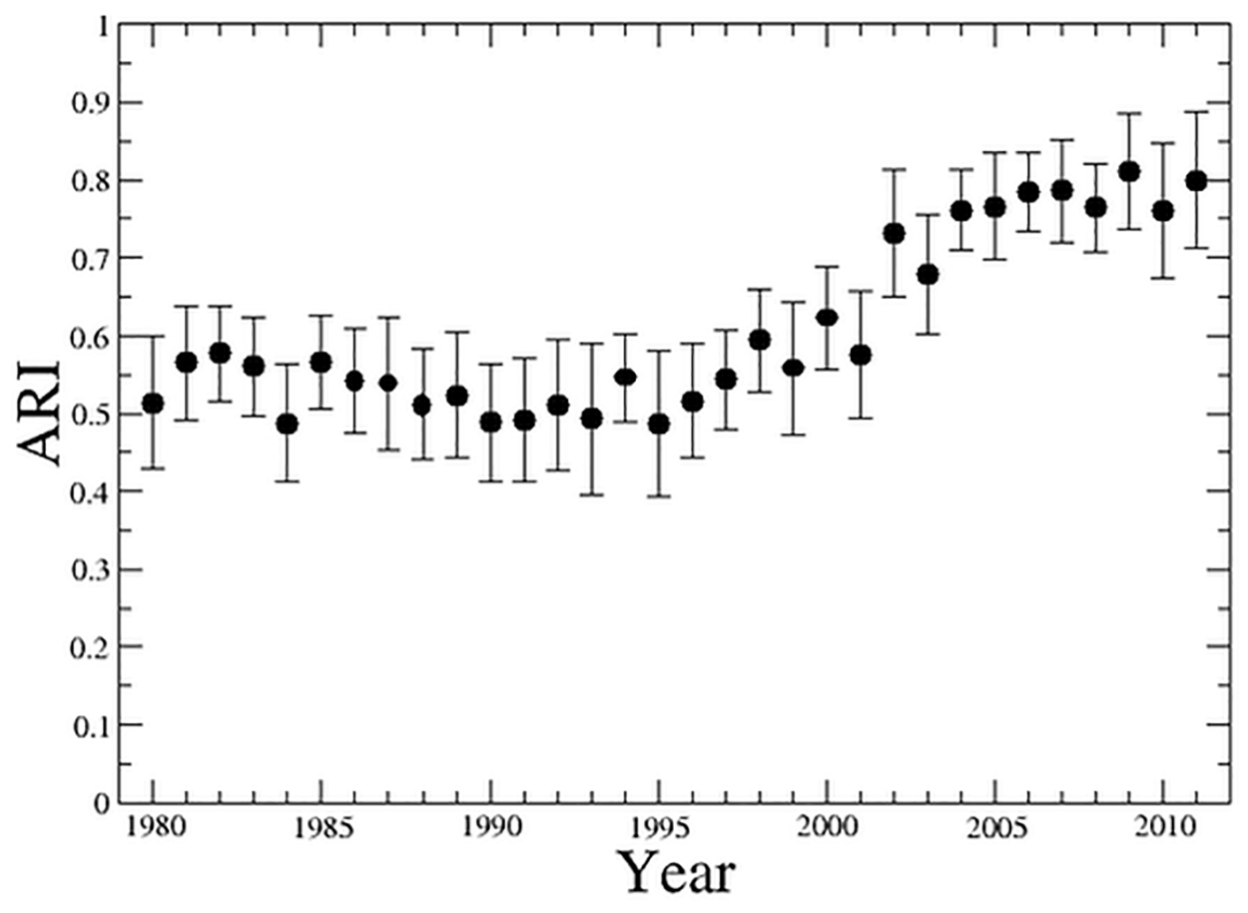
Mean value of the Adjusted Rand Index (ARI) computed between all pairs of partitions obtained in the 20 independent runs of the BRIM algorithm for each calendar year. Error bars indicate one standard deviation.

To quantitatively evaluate the differences which are present among the partitions obtained in the 20 independent runs performed for each calendar year, we evaluate the adjusted Rand index (ARI) [15] among all the pairs of partitions of the 20 runs. In Fig 1 we show the mean value of the adjusted Rand index as a function of the calendar year. The mean value is computed for the set of 190 distinct pairs of partitions obtained from the 20 independent runs of the BRIM computed each calendar year. The error bars are one standard deviation. The adjusted Rand index is close to 0.55 from 1980 to 1995 and increases to approximately 0.8 in the time interval from 2000 to 2011. A value of the adjusted Rand index equals to one would indicate a perfect overlap of the two compared partitions whereas a value close to zero would indicate a random distribution of the nodes into the partitions. Therefore mean values ranging from 0.5 to 0.8 indicate that the different runs provide different partitions. However, the different partitions obtained retain a high fraction of nodes within the same communities. Moreover the degree of overlap of the partitions obtained by independent runs increases in the second half of the investigated time period.

**Fig 2:**
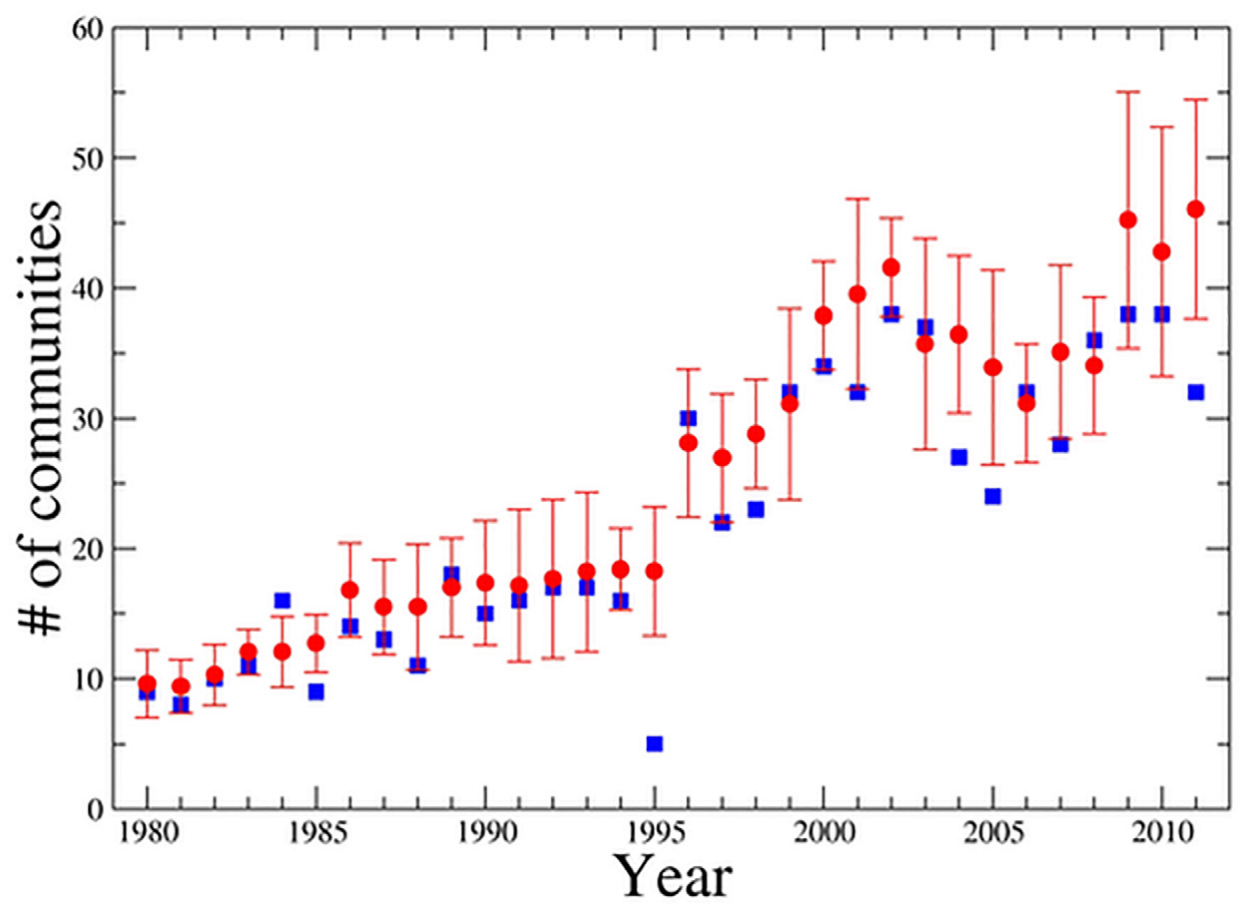
Mean value (red circle symbols) of the number of communities obtained by applying the BRIM algorithm to the bipartite credit system bank-firm for each calendar year of the time interval 1980–2011. The mean value is obtained by averaging the number of communities observed in the 20 independent runs performed by using random initial conditions. Error bars indicate one standard deviation. The blue symbols indicate the number of communities obtained in the partition of best modularity.

To provide an indication of the differences observed among the partitions obtained in independent runs, in Fig 2 we show the time evolution of the average number of communities (red symbol) and its standard deviation obtained for each investigated year. In the figure, we also show the number of communities (blue square symbol) of the partition with the highest modularity for each year. The figure presents an overall gradual increase of the number of communities over time. The figure also shows the presence of an abrupt change of the average number of communities that it is observed between 1995 and 1996. The reason for this abrupt change is that starting from 1996 the database is including OTC firms and therefore comprises a larger set of firms. It is worth noting that in spite of that the mean value of the adjusted Rand index (see Fig 1) is not affected by the change of the size of the investigated system.

## Results

### Time evolution of communities

The communities detected by using the BRIM algorithm and discussed in the previous section are obtained year by year. It is therefore of interest to properly put communities detected on a given year in relation with communities detected in the following year. A time evolution of the communities can be detected by considering what are the communities of year *t* + 1 in which one detects an over-expressed amount of elements of a given community of year *t*. The community detection procedure has a certain degree of stochasticity and degeneracy with respect to small differences of the fitness measure and therefore the membership of an element into a given community might also just be due to chance. We therefore need a method detecting over-expression of the same composition in communities of two successive years that is based on a carefully devised statistical procedure which is robust to the size heterogeneity of the different communities.

We propose the following method. Suppose that in period *t* there are *N*_*t*_ communities $C_{i}^{t}$, *i* = 1, ⋯, *N*_*t*_ and in period *t* + 1 there are *N*_*t*+1_ other communities $C_{j}^{t+1}$, *j* = 1, ⋯, *N*_*t*+1_. For all the $C_{i}^{t}$ communities of period *t* we search amongst all *N*_*t*+1_ communities of period *t* + 1 which communities $C_{j}^{t+1}$ have an over-represented composition of elements also present in a community at time *t*. Specifically, let us call $n_{i}^{t}$ the number of elements in $C_{i}^{t}$, $n_{j}^{t+1}$ the number of elements in $C_{j}^{t+1}$ and $n_{ij}^{t,t+1}$ the number of common element between $C_{i}^{t}$ and $C_{j}^{t+1}$. Let us call *N* ^*t*,*t*+1^ the number of distinct vertices in the two consecutive periods *t* and *t* + 1. The probability that $n_{ij}^{t,t+1}$ is observed by chance is given by the hypergeometric distribution $H(n_{ij}^{t,t+1}|N^{t,t+1},n_{i}^{t},n_{j}^{t+1})$ where:

$$\begin{array}{c} H\left(X|N,M,K\right)=\frac{\left(\begin{array}{c} M\\ X \end{array})(\begin{array}{c} N-M\\ K-X \end{array}\right)}{\left(\begin{array}{c} N\\ K \end{array}\right)}. \end{array}$$ (1)

Therefore for each pair of communities we can compute a *p*-value

$$\begin{array}{c} p_{ij}^{t,t+1}=1-\sum_{x=0}^{n_{ij}^{t,t+1}-1}H\left(x|N^{t,t+1},n_{i}^{t},n_{j}^{t+1}\right). \end{array}$$ (2)

After setting the appropriate *p*-value threshold *p*_*t*_, the above methodology gives us a way to select the communities in year *t* + 1 that are linked to a given community in year *t* in a statistically robust way.

To avoid the presence of false positive, the *p*-value threshold must be corrected to take into account that we are performing a multiple hypothesis test comparison. Indeed, for each pair of consecutive periods we perform the test *N*_*t*_ ⋅ *N*_*t*+1_ times against the null hypothesis of random distribution of elements among two partitions of communities of consecutive periods. Moreover we perform these tests for all pairs of consecutive years in our dataset, i.e., from 1980 to 2011. The most restrictive multiple hypothesis test correction is the Bonferroni correction, which prescribes that the modified *p*-value threshold *p*_*B*_ is:

$$\begin{array}{c} p_{B}=p_{t}/(\sum_{t=1980}^{2011-1}N_{t}\cdot N_{t+1}). \end{array}$$ (3)

In the present investigation we have set *p*_*t*_ = 0.01.

To better understand the key aspects of our method it is worth comparing the results of the statistical validation procedure with a simple indicator as the transition probability. Let us define the transition probability for community $C_{i}^{t}$ to community $C_{j}^{t+1}$ as the ratio between the number $n_{ij}^{t,t+1}$ of elements of $C_{i}^{t}$ also present in $C_{j}^{t+1}$ divided by the number $n_{i}^{t}$ of elements in $C_{i}^{t}$, i.e. $t_{p}=n_{ij}^{t,t+1}/n_{i}^{t}$.

**Fig 3:**
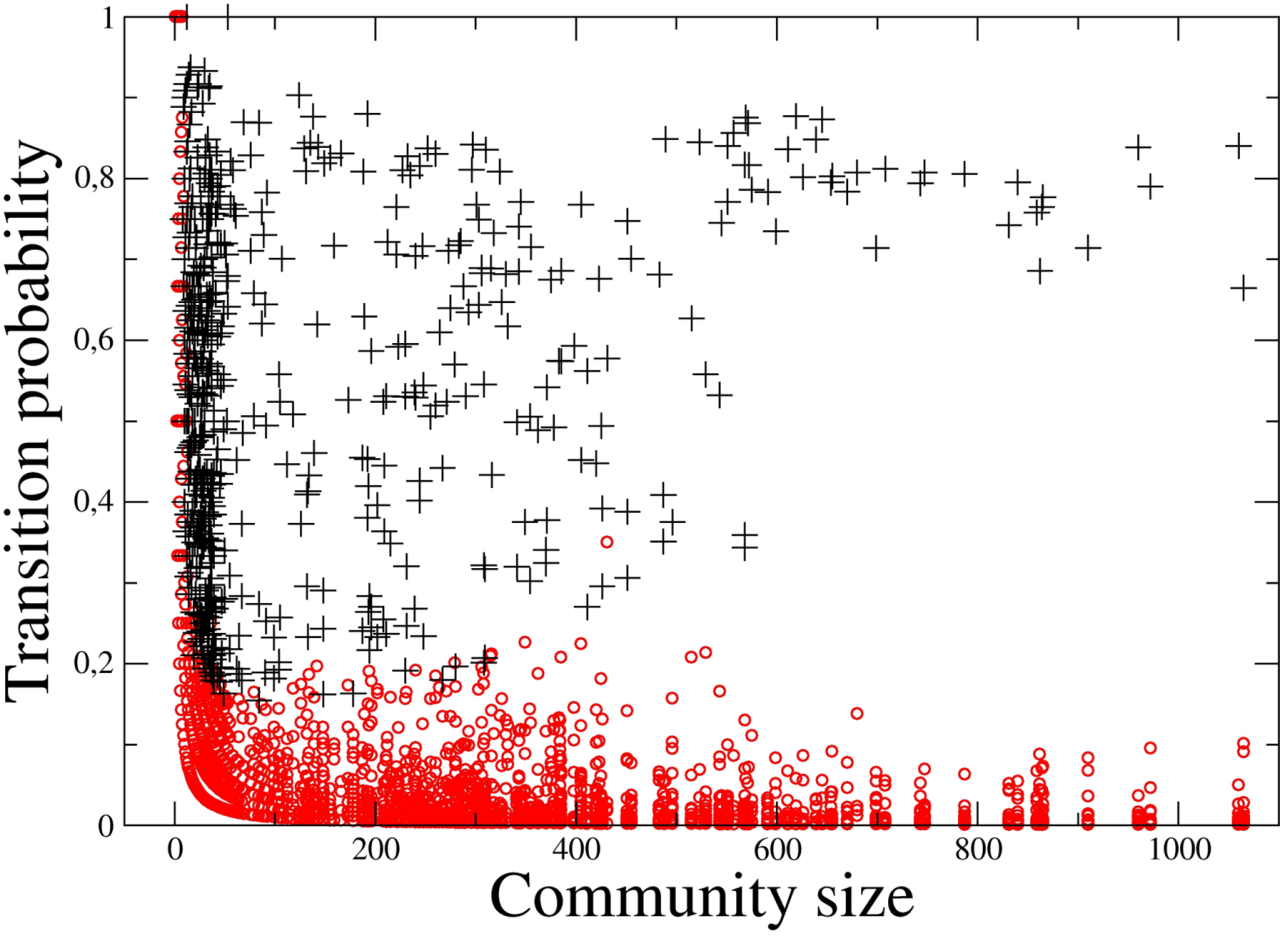
Scatter plot of the transition probability versus the size of the starting community. Each symbol refers to each pair of communities of successive years. Different symbols describe whether the transition from the starting community (at year t) to another community (at year t+1) was validated (black crosses) or not-validated (red circles).

In Fig 3 we show the scatter plot of the transition probability versus the size $n_{i}^{t}$ of the starting community. Each symbol refers to each pair of communities of successive years. Different symbols describe whether the transition from the starting community (at year t) to another community (at year t+1) was validated (black crosses) or not-validated (red circles). For communities with approximately more than 10 elements, the scatter plot shows that our method roughly selects pairs of successive communities of all sizes with transition probability higher or equal to approximately 0.2. The transitions of communities starting with a number of elements smaller or equal to 10 are usually not validated. Along the interface of these two regimes the transitions may or may not be validated for the same value of the transition probability depending on the size of the arriving community. In other words, our statistical validation technique allows us to detect in a coherent way pairs of evolving community both for small and for large starting communities.

**Fig 4:**
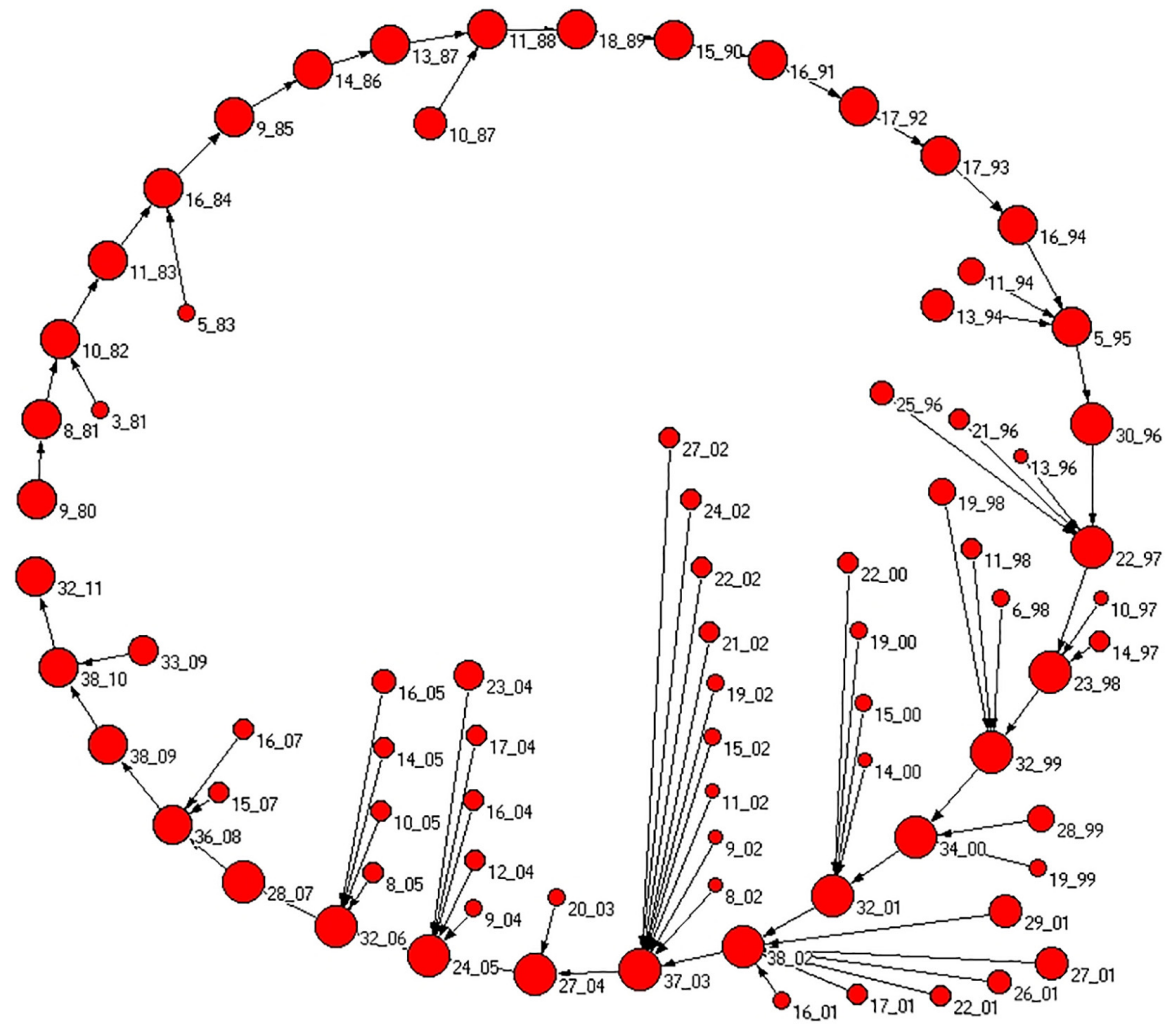
Graphical representation of the interrelationship of communities detected in successive years. The figure shows only the statistical validations observed starting from the largest community of 1980 which is labeled as 9_80 and considering validation between all pairs of communities observed for each pair of successive years (the label of each community is given by a numerical index valid for each specific year which is chosen by ranking the size of the communities detected in that year together with the last two digits of the calendar year. For example 9_80 is the community labeled as 9 of the 9 communities detected in 1980). The size of the vertex symbol is proportional to the logarithm of the size of the community. For any year, the largest community always evolves into the largest community of the successive year.

In Fig 4 we show a graphical representation of the interrelationships of communities that are statistically validated in successive years. The graphical representation is the time evolution of the biggest community of 1980 (labeled as 9_80). The size of each vertex is proportional to the logarithm of the size of the community. The statistical validation procedure shows that the largest community of year *t* evolves into the largest community of year *t* + 1 for all the investigated years. In addition to this primary channel of community evolution we also detect that in some years other smaller communities merge part of them into the largest one (this process is more pronounced during the years 2000, 2001 and 2002). For the sake of clarity, among the communities merging into the largest community, only communities at one year distance from the largest community of each year are shown in the figure. In the following section we will investigate the over-expression of the attributes characterizing the elements of the largest community observed in each calendar year.

**Fig 5:**
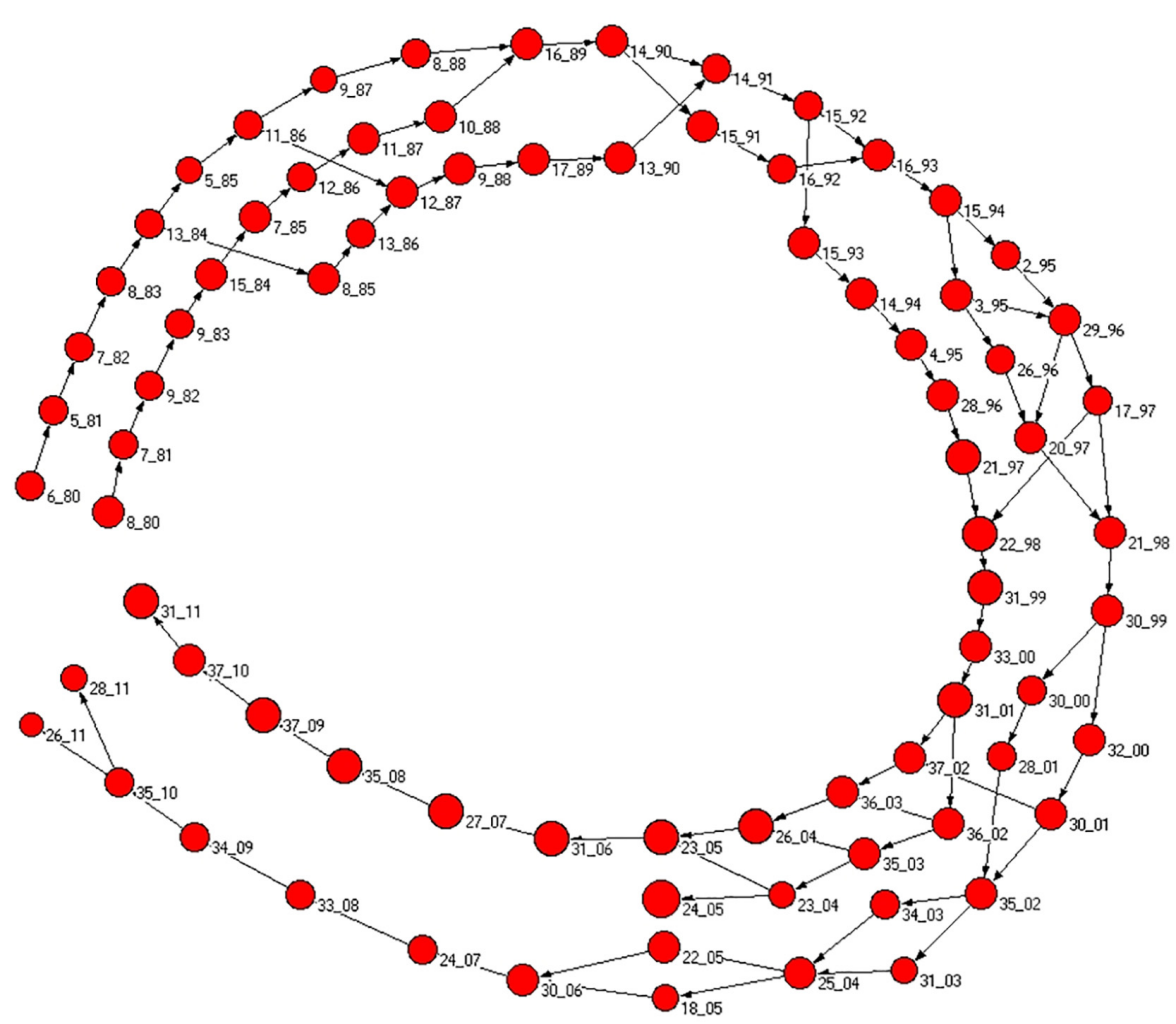
Graphical representation of the interrelationship of communities detected in successive years. The figure shows only the statistical validations observed starting from the second and the third largest communities of 1980 (labeled as 6_80 and 8_80) and considering validation between all pairs of communities observed for each pair of successive years. Only statistically validated directed connection from each community to the ones of the successive year are shown. The incoming validated connection from other communities of the previous year are not shown to make the figure readable. The size of the vertex symbol is proportional to the logarithm of the size of the community. The figure shows that the statistical validation of the communities presents the evolution of three main branches. One of these branches merges into the evolution of the largest one in 2005 (see the evolution of 23_04 in 24_05).

In Fig 5 we track the evolution of the second and the third largest communities of 1980 (labeled as 6_80 and 8_80). In this case the evolution of these communities presents three main branches shown in the figure as parallel evolving branches. However, splitting and coalescence of the branches are observed over time. In this figure we show only “forward” community evolution, i.e., we show all the validated relationships between communities shown at year *t* with communities at year *t* + 1 but, differently than in Fig 4 we do not show validated relationships between communities at year *t* + 1 and communities at year *t* different from the one already shown in the figure. The additional incoming validated connections from other communities of the previous year are not shown to make the figure readable. As in Fig 4, the size of each vertex symbol is proportional to the logarithm of the size of the community. The over-expression of attributes of elements belonging to the communities of the three main branches will be discussed in the following Section.

**Fig 6:**
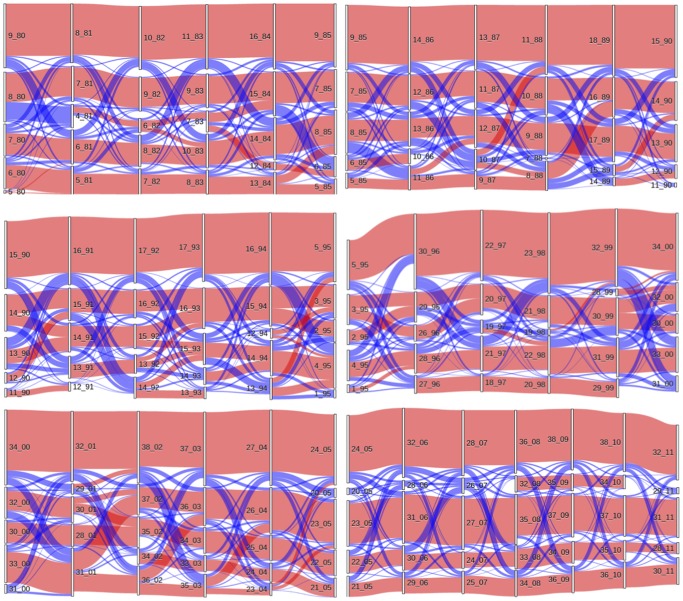
Alluvial diagram of the top 5 biggest communities. Each panel except the last one at the bottom right is showing a five year period. The different periods of time are 1980–1985 (top left), 1985–1990 (top right), 1990–1995 (middle left), 1995–2000 (middle right), 2000–2005 (bottom left), 2005–2011 (bottom right) respectively. Each year is associated with the vertical yellow segments. Different segments refer to different communities. The fluxes shown in red colors are indicating statistically validated relationships between successive communities whereas fluxes shown in blue colors are not validated.

One way to visualize time evolving communities is the one using so-called alluvial diagrams [16]. These diagrams provide a visualization of the flux of elements from communities at step *t* to communities at step *t* + 1. In Fig 6 we show the alluvial diagram for the five biggest communities of our system. The alluvial diagram confirms that there is a high level of persistence of the community composition over the years and that communities from time to time evolve in size, merge or split. We want to point out that alluvial diagrams are not equivalent to our Figs 4 and 5 showing the statistically validated transitions from starting communities to arriving communities. Indeed alluvial diagrams show all transitions and treat all transition on the same basis. To make this point clear, in the alluvial diagram of Fig 6 we have labeled the transitions with two different colors (red and blue). The red color is used for validated transitions and blue color is used for not validated transitions. By inspecting the alluvial diagram of Fig 6 one can follow the time evolution of most of the validated transitions between communities which are shown in Figs 4 and 5.

The color pattern of Fig 6 shows that not all the community transitions can be considered equivalent on a statistical basis. In fact some of them are not consistent with a null hypothesis of random pairing taking into account the heterogeneity in size of the communities (red fluxes) and some are (blue fluxes). We propose to integrate the alluvial diagrams with the results of our statistical validation procedure so that statistically robust transitions can be tracked over time more easily.

### Over-expression of attributes

The identification of bank-firm communities and the statistical validation of their time evolution provides the basis for detecting and understanding the networked structure of the credit system and its time evolution. A further step is to look for information characterizing the obtained communities and their time evolution. In other words it is important to characterize the communities in terms of attributes over-expressed by the elements belonging to the same communities with respect to an appropriate random null hypothesis. The method used here was introduce in [4]. It should be noted that the null hypothesis takes into account the heterogeneity of the tested attributes and therefore the over-expressed attributes are not necessarily the most common ones in each community, but rather are those whose frequency in the community is over-expressed with respect to a null hypothesis taking into account the heterogeneity of attributes. In our analysis we account for multiple hypothesis test correction by using the Bonferroni correction.

The metadata available for the characterization of firms and banks allows us to identify the economic sector and the prefecture of the main office of firms and the type of bank. The summary statistics for each year and for each attribute is provided in the supporting information (S1–S9 Tables). In Table 1 we summarize the over-expressed attributes observed for the time evolution of the largest community (see Fig 4). In the Table we provide the calendar year, the number of banks in the community, the number of firms in the community, the over-expressed (i) prefectures where firms are located, (ii) economic sectors of the firms, and (iii) types of banks. We notice that the type of bank over-expressed in this community is the type labeled as “City banks” for the majority of the investigated years. These banks are large commercial banks operating in the entire country. The fact that the over-expression of “City banks” is not observed after 2005 does not mean that the role of City banks is no more present in those years. In fact also for those years we detect a significant number of City banks in the considered community. The reason why this bank category start to be not over-expressed lays in the fact that the number of “City banks” is declining over time (primarily due to merging, see S1 Table for the number of city banks active each year) and the validation procedure is conducted at the most severe level of multiple hypothesis test correction. In fact the Bonferroni threshold used to validate the over-expression is set to 0.01/*R*_*t*_ where 0.01 is the univariate threshold and *R*_*t*_ = (*N*_*S*_ + *N*_*P*_ + *N*_*B*_) ⋅ *N*_*t*_ is the total number of tests done in the statistical validation of communities of the year *t*. More specifically, *N*_*S*_ is the number of distinct economic sectors, *N*_*P*_ is the number of distinct Japanese prefectures, *N*_*B*_ is the number of types of banks, and *N*_*t*_ is the number of communities detected at year *t*. In this way, we minimize the number of false positive but unavoidably increase the number of false negative.

**Table 1:** Summary of information about the largest community detected by the BRIM algorithm in each calendar year. In the table for each community we report the year, the number of banks, the number of firms, the over-expressed Japanese prefecture of firms (the information is provided in terms of the standard 2 digit code), the over-expressed economic sector, and the over-expressed bank type. The 2 digit prefecture code is the one of Japan’s International Organization for Standardization, and it can be found online at the web page Prefectures of Japan in Wikipedia. According to the 2 digit prefecture code we have: 13 Tokyo and 14 Kanagawa. The over-expressed economic sectors are Electric and Electronic Equipments (EEE), Services (S), and Wholesale trade (WT). The over-expressed type of bank is “city banks” (CB).

Year	banks	firms	prefecture	sector	bank type	Year	banks	firms	prefecture	sector	bank type
1980	23	557	14	EEE	CB	1996	18	975	13 14	S	CB
1981	18	514	14	EEE	CB	1997	14	971	13 14	S	CB
1982	21	534	14	–	CB	1998	16	1069	13 14	S	CB
1983	21	560	14	–	CB	1999	14	1104	13 14	S, WT	CB
1984	16	561	14	EEE	CB	2000	11	959	13 14	S, WT	CB
1985	20	561	14	EEE	CB	2001	9	865	13	S, WT	CB
1986	18	564	–	–	CB	2002	9	917	13	S, WT	CB
1987	16	555	–	–	CB	2003	8	891	13	S, WT	CB
1988	20	611	–	EEE	CB	2004	11	912	13	S, WT	–
1989	19	613	14	EEE	CB	2005	6	905	13	S, WT	CB
1990	21	643	13	–	CB	2006	8	902	13 14	S, WT	–
1991	20	664	–	S	CB	2007	6	857	13 14	S, WT	–
1992	19	614	13 14	–	CB	2008	7	813	13 14	S, WT	–
1993	15	638	13	EEE	CB	2009	11	787	13 14	S, WT	–
1994	18	670	13	–	CB	2010	11	748	13 14	S, WT	–
1995	20	691	–	–	CB	2011	8	725	13 14	S, WT	–

The over-expressed prefectures are the prefectures of Kanagawa (14) and Tokyo (13), i.e. two prefectures of the so-called greater Tokyo area. The Table also shows the over-expression of the main economic sectors of the firms belonging to the community. The over-expressed economic sectors are Electric and electronic equipment (EEE) for the time period 1980–1993, and Services (S) and Wholesale trade (WT) for the time period 1996–2011.

In summary the credit relationships preferentially detected in the largest community change type of over-expressed economic sector during the years 1995–1996. On the other hand, the over-expression of bank type and geographical location remains essentially the same before and after 1996.

To rule out the possibility that the changes observed in the over-expression of economic sectors are due to the 1996 inclusion of the OTC firms we have repeated the community detection and the over-expression characterization by considering the listed firms and the OTC firms separately. Information about the number and attributes of listed and OTC firms is given in the supporting information (S7–S9 Tables).

The results of the separate analyses are presented in Table 2. The results show that the economic sector of Services is over-expressed in the largest community both for listed and OTC firms. For both sets the over-expression of city banks is also confirmed. The number of over-expressions detected in the separate sets is less than in the case of the complete set (see Table 1) but over-expression of Services is clearly detected in both sets ruling out the possibility that the transition from the over-expression of the sector Electric and Electronic Equipment to the over-expression of the sector Services was due to the inclusion of the OTC firms. Concerning the Wholesale trade economic sector that was over-expressed in the complete set from 1999 to 2011, we observe over-expression only in 2003 in the OTC subset. We believe that the absence of the WT validation is due to the too restrictive multiple hypothesis test correction we are using. In fact for the years from 1999 to 2011 we observe that the p-values for this attribute are less than 0.001 in 6 cases for the listed firms and in 3 cases for the OTC firms again supporting the conclusion that the over-expression of the WT sector in the complete set is not due to the inclusion of the OTC firms.

**Table 2:** Summary of information about the largest community detected by the BRIM algorithm in each calendar year for the sets of data considering only the credit relationships with the subset of listed firms (left part of the table) or only the credit relationships with the subset of OTC firms (right part of the table). In the table for each community we report the year, the number of banks, the number of firms, the over-expressed Japanese prefecture of firms (the information is provided in terms of the standard 2 digit code), the over-expressed economic sector, and the over-expressed bank type. According to the 2 digit prefecture code we have: 13 Tokyo, 14 Kanagawa and 27 Osaka. The over-expressed economic sectors are Services (S), and Wholesale trade (WT). The over-expressed type of bank is “city banks” (CB).

Listed firms						OTC firms					
Year	banks	firms	prefecture	sector	bank type	Year	banks	firms	prefecture	sector	bank type
1996	20	710	14	S	CB	1996	11	207	13	–	–
1997	19	719	–	–	CB	1997	12	260	13	–	CB
1998	20	682	14	–	CB	1998	12	290	13	–	CB
1999	16	714	–	–	CB	1999	12	271	13	S	CB
2000	17	613	–	–	CB	2000	11	299	13	–	CB
2001	14	524	13 14	–	–	2001	7	267	13	–	CB
2002	10	572	13 14	–	CB	2002	10	378	13 14	–	–
2003	10	482	–	–	–	2003	13	401	13	S, WT	–
2004	11	471	–	S	–	2004	5	338	13	S	CB
2005	9	494	27	S	CB	2005	8	373	13	S	CB
2006	12	463	27	S	–	2006	8	396	13	S	–
2007	6	449	14	S	–	2007	8	401	13	S	CB
2008	9	408	27	S	–	2008	11	389	13	S	–
2009	16	411	–	S	–	2009	4	223	13	S	–
2010	8	519	13	–	–	2010	4	242	13	–	–
2011	8	385	14	–	–	2011	10	295	13	–	–

In Fig 5 we have shown the time evolution of the second and third largest communities of 1980. In this case we observe an evolution of the communities that on average presents three main branches characterized by the over-expression of several Japanese prefectures, economic sectors and type of banks. All over-expression are summarized in Table 3 where we note three main branches of community. The first one starts in 1980 and last until 2011 (see the first column of Table 3). It presents over-expression of firms of economic sectors Utilities electric (U) and Credit Leasing (L). Until 2001 the over-expressed banks are Life-insurance (LI) banks and Insurance banks (IB). The over-expression of Utilities electric is observed until 2000. Starting from 2000 only firms belonging to the Credit Leasing economic sector are over-expressed. For this branch of communities the geographical location of firms shows that the Japanese prefectures of Tokyo (labeled as 13), Hiroshima (34) and Fukuoka (40) are over-expressed in several years. During the most recent years several prefectures of the western part of Japan (e.g. prefectures labeled as 28 (Hyogo), 33 (Okayama), 34, 37 (Kagawa) and 40) are over-expressed.

**Table 3:** Summary of information about the evolution of a few large communities detected by the BRIM algorithm in each calendar year. The evolution follows the scheme shown in Fig 5. In the table for each community we report the code of the community (id_year), the number of banks, the number of firms, the over-expressed Japanese prefecture of firms (the information is provided in terms of the standard 2 digit code), the over-expressed economic sector of firms, and the over-expressed bank type. The over-expressed economic sectors are Construction (C), Credit Leasing (CL), Chemicals (Ch), Electric and Electronic Equipments (EEE), Motor parts (MV), Railroad Transportation (RT), Sea Transportation (ST), Services (S), Utilities electric (U) and Wholesale trade (WT). The over-expressed type of bank are “city banks” (CB), Life-insurance banks (LI), Regional banks (RB), Insurance banks (IB), and Second regional banks (SR).

comm.	*N*_*b*_	*N*_*f*_	pref.	sector	B.t.	comm.	*N*_*b*_	*N*_*f*_	pref.	sector	B.t.	comm.	*N*_*b*_	*N*_*f*_	pref.	sector	B.t.
6_80	45	241	–	–	LI IB	8_80	128	305	–	–	RB SR						
5_81	47	201	–	RT U	LI IB	7_81	97	214	13	C	RB						
7_82	49	194	–	U	LI IB	9_82	89	208	13	C	RB						
8_83	46	166	13	U	LI IB	9_93	85	211	–	C	RB						
13_84	47	146	–	U	LI IB	15_84	111	277	–	–	RB						
5_85	41	102	–	–	IB	7_85	85	260	–	–	RB	8_85	29	381	–	RT	–
11_86	48	164	–	U	LI IB	12_86	80	228	–	C	RB	13_86	21	292	–	–	–
9_87	52	108	13	CL U	LI IB	11_87	89	235	40	C	RB	12_87	16	319	–	RT	–
8_88	58	203	13	U	LI IB	10_88	113	314	40	C	RB SR	9_88	21	339	–	RT	
16_89	121	225	34 40	CL U	IB							17_89	21	367	–	RT	–
14_90	135	220	13	CL	–							13_90	15	336	–	RT	–
14_91	51	237	–	CL U	LI IB	15_91	93	223	34 40	C	RB						
15_92	52	188	13	CL	LI IB	16_92	78	199	34 40	C	–						
16_93	135	273	13 40	CL								15_93	11	309	–	RT	–
15_94	126	246	13	CL U	IB							14_94	13	322	–	RT	–
2_95	57	169	–	CL U	LI IB	3_95	102	325	34 40	C	RB	4_95	20	486	–	Ch RT ST	–
29_96	45	241	–	–	LI IB	26_96	128	305	–	–	RB SR	28_96					
17_97	71	149	13	CL U	IB	20_97	72	361	1 15 34 40	C	RB	21_97	17	524	–	Ch MV RT	–
21_98	119	342	34 40	C CL	–							22_98	34	499	–	Ch RT	LI
30_99	119	377	34 40	-C CL U	–							31_99	24	550	26 27	Ch	–
32_00	92	345	10 13	CL	IB	30_00	42	226	33 34 38 40 43 46	–	–	33_00	19	477	26 27	Ch	
30_01	93	232	7	C CL	IB	28_01	34	228	33 34 35 37 38 40	–	–	31_01	16	574	14	Ch	–
35_02	82	278	34 37 40	–	–	36_02	11	379	–	–	–	37_02	26	383	–	–	–
34_03	69	241	27 28 33 34 37	CL	–	35_03	8	326	13	–	–	36_03	26	450	22	–	LI
31_03	21	110	40 46	–	–												
25_04	85	338	33 34 37 40	CL	–	23_04	6	151	14	–	–	26_04	28	550	13	Ch	–
22_05	84	310	1 28 33 34 37	CL	–	24_05	6	905	13	S WT	CB	23_05	26	602	15	RT	LI
18_05	18	109	40 46	–	–												
30_06	71	306	28 33 34 37 40	CL	–							31_06	23	646	13	RT	LI
24_07	27	174	34 35 40 46	–	–							27_07	24	662	13	Ch RT	–
33_08	51	262	28 33 34 37 40	–	–							35_08	30	595	13	CL RT	–
34_09	34	225	28 33 34 37 40	–	–							37_09	29	546	13	–	LI
35_10	42	210	28 33 34 37 40	–	–							37_10	22	483	13	RT	LI
28_11	17	154	28 33 34 37	–	–							31_11	23	514	13	RT	–
26_11	18	89	40 46	–	–												

The second branch starts in 1980 and ends approximately in 2005 (see the second column of Table 3). This second branch presents over-expression of firms of the Construction (C) economic sector and of the Regional banks (RB) and occasionally of the Second regional banks (SR). The geographical over-expression points out Japanese prefectures of Hiroshima and Fukuoka and of Tokyo in a few cases.

The third branch starts in 1985 and ends in 2011 (see the third column of Table 3). In this last case the branch presents persistent over-expression of firms of the Railroad Transportation (RT) and Chemicals (Ch) sectors. An over-expression of banks classified as Life-insurance banks (LI) is observed after 1997. The geographical over-expression mainly involves the prefecture of Tokyo especially during the most recent years.

In summary we observe three distinct branches well characterized over time by a rather stable over-expressions of economic sector and type of banks. Also the over-expression of the regional location of firms, although less stable than the ones of the economic sector and of the type of bank, shows a high degree of persistence over time. The communities of banks and firms detected by the BRIM are able to detect a networked nature of the Japanese credit market with a time scale of the dynamics of the communities covering several years.

## Discussion

In our study we analyze the time evolution of the bank-firm credit relationships in Japan over a period of time of 32 years. The analysis is performed on the bank-firm bipartite network observed yearly. For each year, we detect communities communities of banks and firms and characterize them with respect to the over-expression of firms’ economic sectors, firms’ Japanese prefectures, and types of banks.

In our study it was crucial to select a community detection algorithm directly working on the bipartite network that is providing communities composed by both types of vertices (banks and firms). The choice of a one-to-one correspondence between banks’ partitions and firms’ partitions also simplify our analysis of joint over-expression of attributes of banks and firms. With this approach we have been able to show the existence of layers of the credit market involving an over-expression of groups of firms characterized by specific (i) economic sectors, (ii) regional locations (prefectures), and (iii) specific types of banks. These empirical observations show that the credit market in Japan is a networked market.

The robustness of our results is shown by the ability of our approach in detecting both the long term stability and the slow dynamics of the detected communities. The time evolving communities have been tracked from each year to the next one by using a newly introduced statistical method able to track the time evolution of communities detected in successive periods of time also in the presence of size heterogeneity of the communities. It is worth noting that our method presently used to track time evolution of communities can also be easily adapted to link communities detected in a multiplex network.

## Supporting Information

S1 Table Summary statistics of the type of bank for the complete set during years 1980–2011. (XLSX)

S2 Table Summary statistics of geographical location attribute of firms for the complete set during years 1980–2011. (XLSX)

S3 Table Summary statistics of economic sector attribute of firms for the complete set during years 1980–2011. (XLSX)

S4 Table Summary statistics of the type of bank for banks providing credit to listed firms during years 1996–2011. (XLSX)

S5 Table Summary statistics of geographical location attribute of listed firms during years 1996–2011. (XLSX)

S6 Table Summary statistics of economic sector attribute of listed firms during years 1996–2011. (XLSX)

S7 Table Summary statistics of the type of bank for banks providing credit to OTC firms during years 1996–2011. (XLSX)

S8 Table Summary statistics of geographical location attribute of OTC firms during years 1996–2011. (XLSX)

S9 Table Summary statistics of economic sector attribute of OTC firms during years 1996–2011. (XLSX)

S10 Table Summary statistics of degree of banks for the complete set during years 1980–2011. (TXT)

S11 Table Summary statistics of degree of firms for the complete set during years 1980–2011. (TXT)

S12 Table Summary statistics of degree of banks providing credit to listed firms during years 1996–2011. (TXT)

S13 Table Summary statistics of degree of listed firms during years 1996–2011. (TXT)

S14 Table Summary statistics of degree of banks providing credit to OTC firms during years 1996–2011. (TXT)

S15 Table Summary statistics of degree of OTC firms during years 1996–2011. (TXT)
